# CRATS: a one-pot CRISPR-Cas12b and RPA combined assay with a temperature switchcong for highly sensitive detection of monkeypox virus: CRATS for highly sensitive detection of monkeypox virus

**DOI:** 10.3724/abbs.2025016

**Published:** 2025-04-02

**Authors:** Guangxi Yu, Yue Wang, Yukang Chen, Jiangyuan Liu, Hongtao Kang, Xiaodong Luan, Song Gao, Pei Wang

**Affiliations:** 1 Jiangsu Key Laboratory of Marine Biological Resources and Environment Co-Innovation Center of Jiangsu Marine Bio-industry Technology Jiangsu Ocean University Lianyungang 222005 China; 2 School of Electronic Engineering Jiangsu Ocean University Lianyungang 222005 China; 3 School of Marine and Biological Engineering Yancheng Teachers University Yancheng 224007 China

CRISPR-Cas nucleases have been extensively used in molecular detections, especially highly sensitive nucleic acid detections [1]. In these detections, Cas nucleases are programmed by the guide RNA to respond to the detection targets and cleave the chemically labelled molecular beacons by the *trans*-cleavage activity to produce the detection signal. To improve sensitivity, nucleic acid amplification technologies are usually introduced to give a pre-amplification of the nucleic acid targets, increasing the detection sensitivity extraordinarily. Polymerase chain reaction (PCR) technology has been used for pre-amplification in laboratories, and isothermal amplification technologies are applied to meet point-of-care testing (POCT) needs because they avoid the use of sophisticated thermal cycling devices. The recombinase polymerase amplification (RPA) technology that amplifies nucleic acid targets isothermally at 37–42°C has been combined with CRISPR-Cas nucleases to establish advantageous nucleic acid detection assays, *e* . *g*., the SHERLOCK, which combines with Cas13a [2], and the DETECTR, which combines with Cas12a [3]. 

It has been challenging to integrate Cas nucleases and RPA in a one-pot reaction system because the cleavage activity of Cas stimulated by even small amounts of the targets can interfere with amplification by digesting the primers or the newly amplified fragments. Thus, many assays based on Cas nucleases and RPA are in a two-step setting, with pre-amplification and Cas cleavage being isolated as two independent procedures. The two-step setting ensures that amplification and cleavage occur under favorable conditions but sacrifices operational convenience and introduces the risk of cross-contamination. In efforts to establish one-pot RPA-Cas assays, many strategies have been applied, including the use of photocontrolled guide RNA to activate the Cas nuclease at a preferred timepoint [4], the use of a suboptimal protospacer adjacent motif (PAM) to suppress Cas activity [5], the generation of dynamic aqueous multiphase with sucrose or glycerol to partially separate the two reactions [6], and extensive optimization of the RPA-CRISPR reaction system to achieve a subtle balance between the two reactions [7]. 

In this study, a one-pot CRISPR-Cas12b and RPA combined assay with a temperature switch (CRATS) was established utilizing the reaction temperature difference between RPA and CRISPR-Cas12b cleavage. The Cas12b used in this study, AaCas12b, is a type V-B CRISPR‒Cas nuclease from *Alicyclobacillus acidiphilus* [8]. It has a bi-lobed architecture consisting of an *α*-helical recognition lobe containing the REC domains and a nuclease lobe containing the WED, RuvC and Nuc domains. As a dual-RNA-guided DNA endonuclease, Cas12b can be guided by a chimeric single-guide (sg) RNA, and its *trans*-cleavage activity is specifically activated by the DNA target and results in nonspecific cleavage of single-stranded (ss) DNA molecules, which can be used to produce detection signals if the ssDNA is appropriately labelled as the molecular beacon. As the temperature for RPA is 37°C and the *trans*-cleavage of Cas12b is active at 60°C, CRATS uses temperature switching to adjust the major on-going reaction in the one-pot system and realizes sequential amplification of the target and cleavage reactions for signal detection. The detection target of this study, monkeypox virus, is an infectious pathogen that has caused the announcement of the Public Health Emergency of International Concern (PHEIC) by the World Health Organization (WHO) twice in recent years ( https://www.who.int/news). In this one-pot CRATS assay, the reaction reagents of CRISPR-Cas12b and RPA are mixed in a single tube. After the addition of the sample containing the detection target, the reaction was carried out at 37°C for 20 min for amplification, followed by 60°C for 20 min for Cas12b cleavage. The fluorescently labelled molecular beacon is cleaved by Cas12b to release the FAM fluorophore from quenching, producing a fluorescence signal that is visualized under blue light ( Figure 1). CRATS shows a high sensitivity of 10 ^0^ copies of the target DNA per reaction and good specificity, providing a novel strategy of temperature switching to integrate CRISPR-Cas and RPA in a one-pot reaction system. Moreover, it provides a POCT-friendly tool for the detection of the important infectious pathogen monkeypox virus.

**Figure 1:**
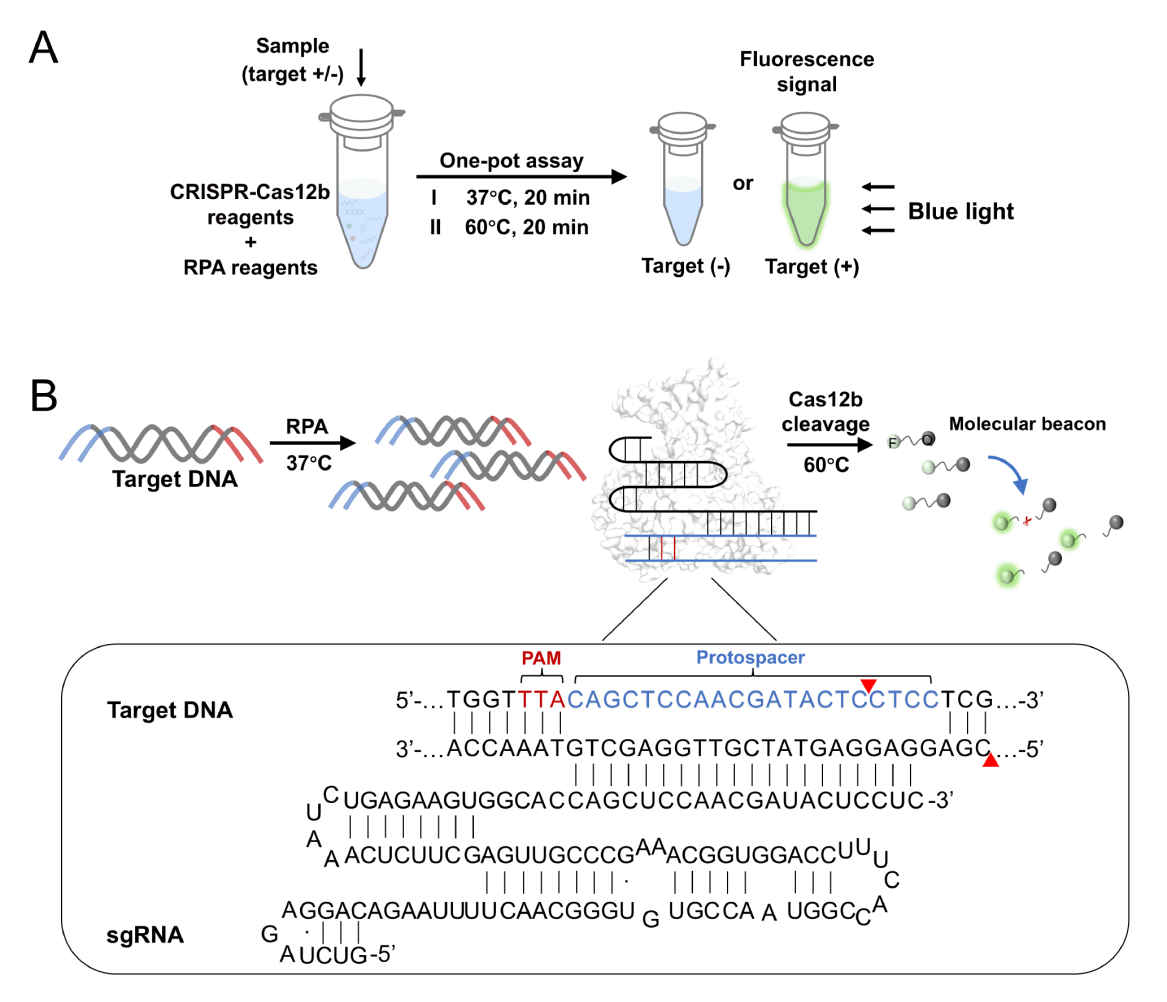
Figure 1 Schematic of the principle of CRATS (A) One-pot procedure. (B) Details of the sequential reactions in one pot. After pre-amplification by RPA, the amplified target DNA, sgRNA, and Cas12b form a cleavage complex that cleaves the molecular beacon upon a temperature switch from 37°C to 60°C. The DNA/RNA sequences are shown in the inset. The PAM (in the 5′-TTN pattern) and the protospacer are indicated. The red triangles indicate the cleavage sites.

CRISPR-Cas12b was recombinantly expressed and purified. The RPA procedure followed the instructions of the RAA nucleic acid amplification reagent (Hangzhou ZC Bio-Sci & Tech Co., Ltd., Hangzhou, China). The RPA primers used in this study followed the sequences of a previous report ( Supplementary Table S1) [9]. Cas12b cleavage was carried out in a 20-μL reaction mixture at 60°C. The sgRNA was designed in reference to a previous report [9] and followed the requirements for appropriate guidance by Cas12b ( Supplementary Table S1). Please see the detailed experimental procedures and sample sources in the Supplementary Materials and Methods. 

The key for CRATS to work is to set the reaction temperature to favor either one of the two reactions in the one-pot system at a time, amplification or Cas12b cleavage. AaCas12b from the thermoacidiphilic bacterium *A*. *acidiphilus* is expected to have the greatest *trans*-cleavage activity at relatively high temperatures. This study confirmed this high-temperature preference by testing Cas12b cleavage on a molecular beacon at 37°C or 60°C. When specifically guided by the sgRNA and activated by the RPA-amplified target fragments, Cas12b showed good cleavage activity at 60°C but very poor cleavage activity at 37°C ( Figure 2A), suggesting that a temperature shift from 37°C to 60°C could transform Cas12b to the working state.

**Figure 2:**
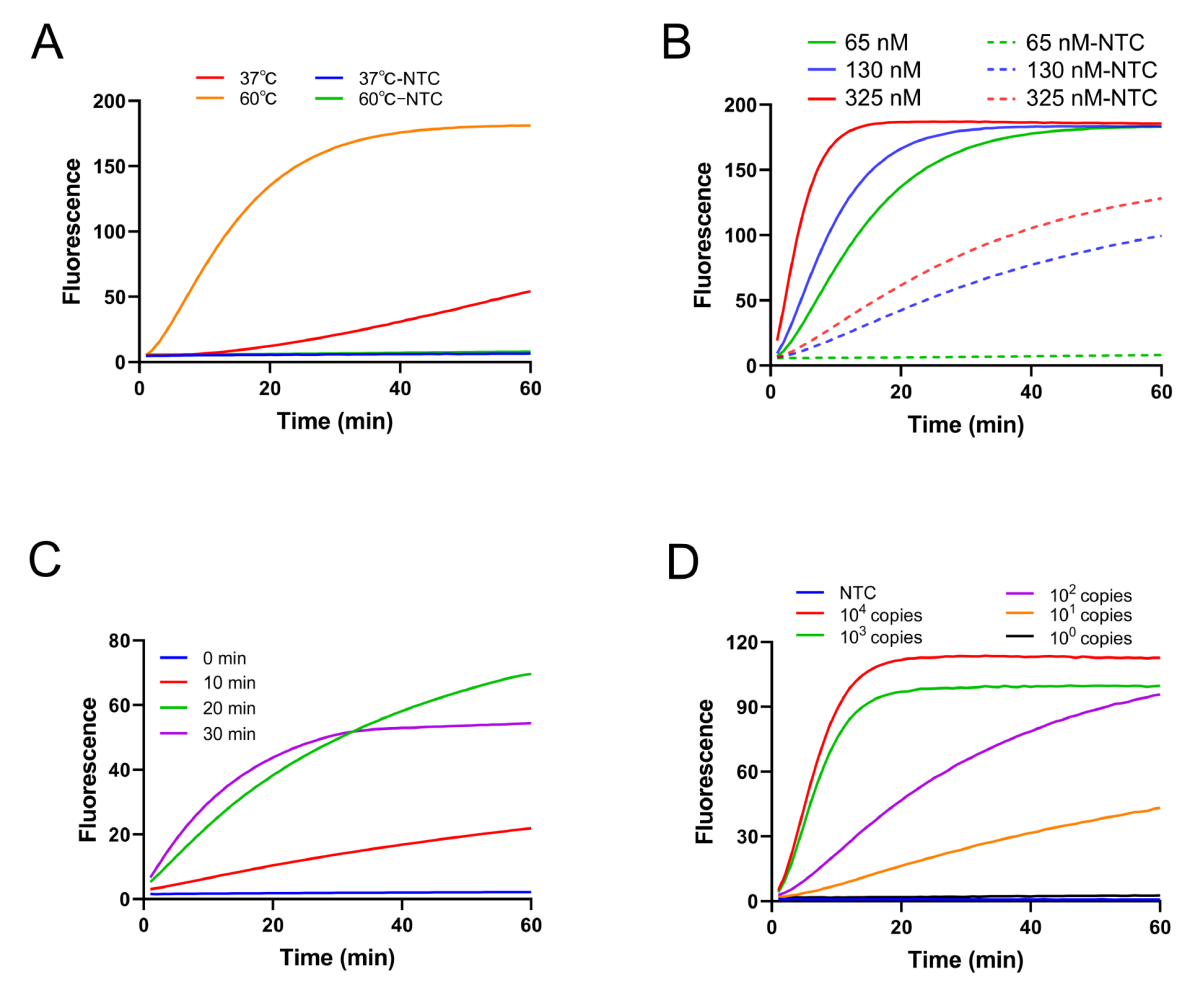
Figure 2 Optimization of the CRATS reaction system (A,B) Temperature and concentration of Cas12b used for cleavage. Fluorescence curves with different colors show the cleavage activities of Cas12b at different temperatures (A) or different amounts of Cas12b (B). The cleavage substrate was the amplicon of a 30-min RPA amplification from 105 copies of the standard DNA. (C) Time for RPA pre-amplification. The different colors indicate different reaction time for the pre-amplification. The cleavage substrate was the amplicon from 102 copies of the standard DNA. (D) Reference sensitivity determined by a two-step procedure. The fluorescence curves with different colors represent the detection signals from different amounts of standard DNA. NTC represents the no-template control. The diagrams represent the results from three independent experiments.

The CRATS procedure includes two reactions, RPA amplification of the target and cleavage of the molecular beacon by Cas12b, and the reaction sequence is expected to be controlled by temperature. To establish the CRATS reaction system, the two reactions were independently optimized. First, the concentration of Cas12b for cleavage was optimized. It was determined that 65 nM Cas12b should be used because higher concentrations did not significantly improve the specific cleavage but instead led to leaking cleavages ( Figure 2B). Second, the time for RPA amplification (preamplification) was optimized. The results showed that pre-amplification for 20 min or longer was sufficient for subsequent cleavage to produce distinct signals ( Figure 2C). A two-step procedure of 20 min preamplification by RPA followed by cleavage of Cas12b at 65 nM, with sample transfer between Cas12b and Cas12b, resulted in as few as 10 ^1^ copies of the target, successfully yielding the detection signal, and cleavage for 20 min was enough to produce an easy-to-read endpoint fluorescence signal ( Figure 2D). Taken together, the one-pot CRATS was constructed on the basis of three key points: (1) In the reaction mixture containing both RPA reagents and Cas12b-related reagents, 65 nM Cas12b should be used; (2) the assay should be carried out with a “20 min at 37°C and 20 min at 60°C” temperature switching sequence; and (3) the sensitivity of 10 ^1^ copies per reaction observed via the two-step procedure can be considered a reference sensitivity for developing the one-pot CRATS. 

The one-pot CRATS reaction mixture was prepared by mixing two premixes, the RPA premix and the Cas12b premix. The RPA premix was prepared by adding 25 μL of A buffer and 4 μL each of the forward and reverse primers (10 μM) to the lyophilized enzyme pellet. The Cas12b premix contained 260 nM purified Cas12b, 2 μM molecular beacon, and 200 nM sgRNA in 1× NEB buffer 2.1 (NEB, Beverly, USA). The 25-μL one-pot CRATS reaction mixture was made by mixing 14.5 μL of RPA premix, 6.25 μL of Cas12b premix, 1.25 μL of B buffer, and 3 μL of the sample. The reaction was carried out at 37°C for 20 min, followed by incubation at 60°C for 20 min in a mini metal bath. The FAM fluorescence signal (excitation wavelength 495 nm) was monitored with a Roche LightCycler 480 II qPCR machine (Roche, Basel, Switzerland). The endpoint fluorescence signal was observed by the naked eye under blue light.

The sensitivity of the one-pot CRATS for the detection of monkeypox virus was tested with standard plasmid DNA. The results revealed a sensitivity of 10 ^0^ copy per reaction ( Figure 3A), making CRATS one of the most sensitive assays for monkeypox virus detection. The improvement in the sensitivity of the one-pot CRATS compared with the two-step procedure was probably due to the one-pot setting, in which all the amplification products spontaneously participated in activating the *trans*-cleavage of Cas12b. The available monkeypox virus detection assays include the qPCR assay recommended by the WHO, which has a sensitivity of several copies per reaction; the isothermal RPA assay, which has a sensitivity of approximately 10–20 copies per reaction [10]; and the combined RPA and CRISPR-Cas12a assays, which have sensitivities as low as a single copy per reaction [9]. Compared with these assays, CRATS is more sensitive.

**Figure 3:**
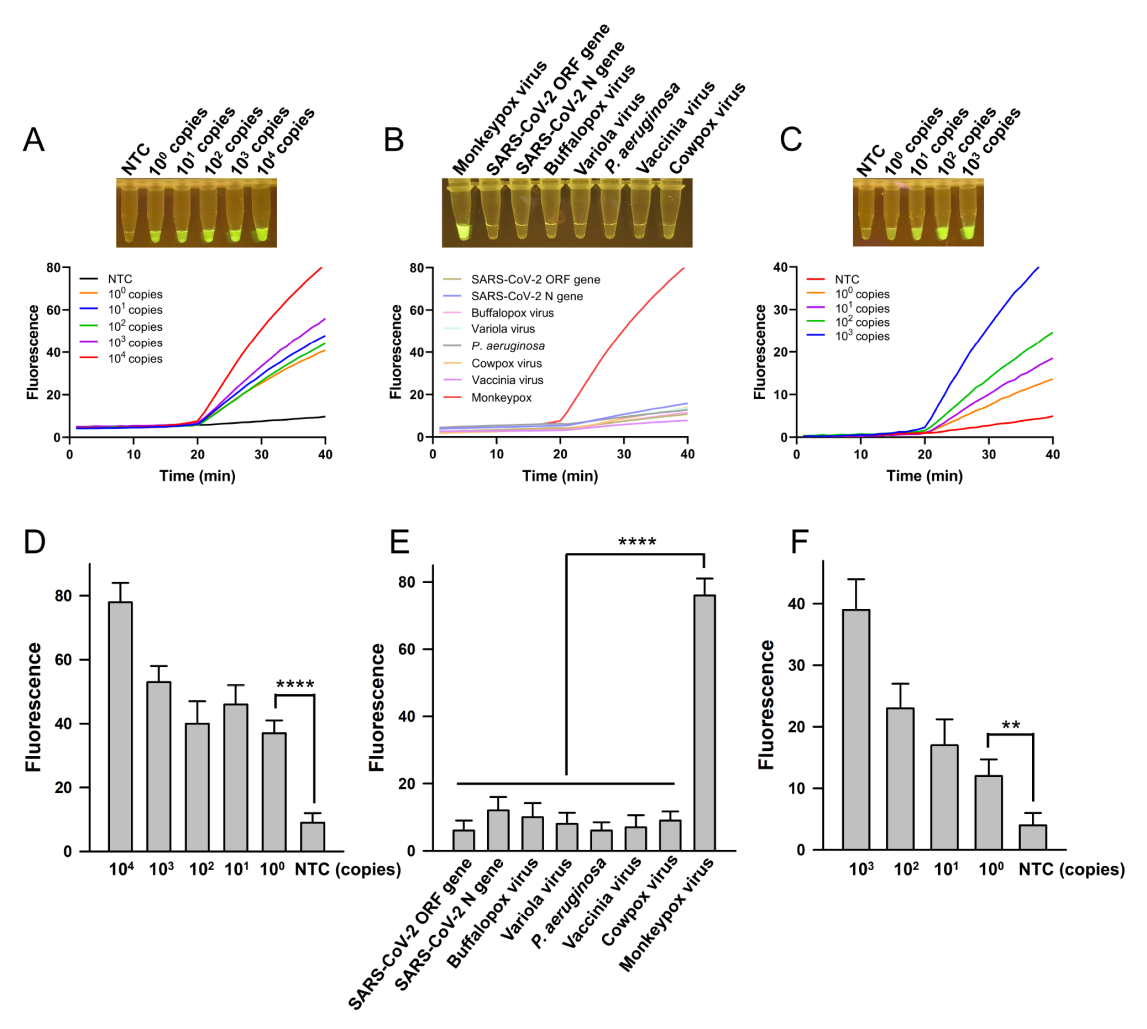
Figure 3 Performance of CRATS (A,D) Sensitivity of CRATS tested with standard DNA. The fluorescence curves with different colors (A) indicate different amounts of the target. The quantification of the signals (values at the 40 min time point of the fluorescence curves) are shown as bar charts (D). (B,E) Specificity of CRATS. The fluorescence curves of different pathogen samples (B) are shown with different colors. The quantified signals are shown as bar charts (E). (C,F) Validation of CRATS with simulated skin samples. Fluorescence curves with different colors (C) show the detection signals of simulated samples containing different amounts of the target. The quantified signals are shown as bar charts (F). The endpoint visualized images of the detection results are shown above the fluorescence curves. NTC represents the no-template control. The error bars represent the standard error of three parallel repeats. **P < 0.01, ****P < 0.0001.

The specificity of one-pot CRATS was confirmed by detecting a series of orthopox viruses, including buffalopox virus, Variola virus, cowpox virus, vaccinia virus, *Pseudomonas aeruginosa*, and the SARS-CoV-2 N and ORF genes. Only the monkeypox virus produced a positive signal ( Figure 3B), suggesting good specificity of CRATS. The detection target used in this study, a portion of the *F3L* gene of monkeypox virus, has already been validated as a specific target for this pathogen [10]. The *F3L* gene encodes an immunomodulatory protein that is highly specific for monkeypox virus and its evolutionary variants, and was selected as the major detection target in pandemics in this decade ( https://www.ndcpa.gov.cn/). The selection of this fragment on the *F3L* gene as the detection target ensured that CRATS was specific, which was further confirmed in this study. 

The one-pot CRATS was validated with simulated skin samples. When these samples containing different amounts of monkeypox pseudovirus were tested, CRATS showed the same good sensitivity as standard plasmid DNA, which was 10 ^0^ copy per reaction ( Figure 3C). These results suggest that CRATS is potentially useful for the clinical diagnosis of monkeypox virus infection. 

Any POCT-friendly assay is valuable for the prevention and control of the monkeypox epidemic. For this purpose, many assays based on isothermal amplification methods and programmable nucleases have been developed [10]. Simple operation, good reliability, acceptable sensitivity, and minimum dependence on laboratory settings are the common goals in developing these assays. CRATS uses a one-pot setting with simple temperature switching on a mini metal bath to complete the detection, and the result is visualized for direct reading, making it a good assay for POCT. Moreover, the sensitivity is the highest among the available assays. This assay has the potential to be further developed with simplified designs and commercialized in the home and community hospital markets. 

In conclusion, a one-pot detection assay, CRATS with temperature-controlled sequential reactions, was established in this study. By temperature switching, the major on-going reaction is appropriately adjusted in the one-pot reaction system, resulting in a change from “active amplification/silent cleavage” to “silent amplification/active cleavage” with simple operation. This not only avoids liquid transfer between reaction mixtures and the related cross-contamination risk but also minimizes the interference between RPA amplification and CRISPR-Cas12b cleavage. Compared with other CRISPR-Cas-based one-pot assays, CRATS utilizes the intrinsic features of RPA and Cas12b, the differently preferred reaction temperatures, which are more reliable and have fewer obstacles when developing a certain detection method, *e*. *g*., extensive optimization of sequences, chemical labels, and/or reaction conditions. Thus, the CRATS strategy is readily applicable to a wide range of nucleic acid detection needs. For the monkeypox virus, this study provides a highly sensitive and POCT-friendly molecular detection method. 

## Supporting information

24819Supplementary_Data

## Supplementary Data

Supplementary data is available at *Acta Biochimica et Biophysica Sinica* online.
