# Supplementary material for: CRATS: a one-pot CRISPR-Cas12b and RPA combined assay with a temperature switchcong for highly sensitive detection of monkeypox virus: CRATS for highly sensitive detection of monkeypox virus
Source: Acta Biochim Biophys Sin (Shanghai). 2025 Apr 2;57(8):1371–5. doi: 10.3724/abbs.2025016 (PMC12368519; doi:10.3724/abbs.2025016)
Supplement: 24819Supplementary_Data [file 24819Supplementary_Data.docx]

**Supplementary Materials and Methods**

**Preparation of CRISPR-Cas12b**

The recombinant expression of CRISPR-Cas12b was carried out with a commercial plasmid, BPK2014-AaCas12b (Enzyme-linked Biotechnology Co. Ltd., Shanghai, China). The plasmid contained the coding sequence for the expression of Cas12b from *Alicyclobacillus acidiphilus* (GenBank No. WP_067623834) with a 6× His tag at the C-terminus. Cas12b was overexpressed in *Escherichia coli* BL21-pLysS cells by IPTG (0.5 mM final) induction for 16 h at 16°C and then purified via Ni-affinity chromatography (AKTAprime Plus; GE Healthcare, Wisconsin, USA). The purified Cas12b was stored in 50 mM Tris, pH 8.0, 200 mM NaCl, 0.1 mM EDTA, 1 mM DTT, and 20% glycerol at −20°C.

**RPA**

The RPA procedure followed the instructions of the RAA nucleic acid amplification reagent (Hangzhou ZC Bio-Sci & Tech Co., Ltd., Hangzhou, China). One lyophilized enzyme pellet was dissolved in 25 μL of A buffer and used for two reactions. For each reaction, 2 μL each of the forward and reverse primers (10 μM), 4.25 μL of ddH_2_O, 3 μL of the sample, and 1.25 μL of B buffer were added. The reaction was carried out at 37°C for 10‒30 min. The RPA primer design considerations included the following: primer length, 30‒35 nt; GC content, 30%‒70%; and amplification product length, 70‒500 bp.

**Cas12b cleavage**

In the 20-μL Cas12b cleavage reaction mixture, 65–325 nM final purified Cas12b, 50 nM final single-guide (sg) RNA, 500 nM final molecular beacon labelled with FAM and BHQ1 at the two ends, and 2 μL of the RPA amplification product were mixed in 1× NEB buffer 2.1 (NEB, Massachusetts, USA). The reaction was carried out at 60°C for 60 min. The FAM fluorescence signal (excitation wavelength 495 nm) was monitored with a Roche LightCycler 480 II qPCR machine (Roche, Basel, Switzerland). For sgRNA preparation, a double-stranded (ds) DNA fragment containing the T7 promoter and the sgRNA sequence was prepared by annealing two reverse-complement single-stranded (ss) DNA fragments (synthesized by General Biology Co., Ltd., Hefei, China). The sgRNA was synthesized via *in vitro* transcription from the dsDNA fragment via a transcription kit (Vazyme Biotech Co., Ltd., Nanjing, China), followed by phenol-chloroform extraction, and was quantified with a Qubit 4 fluorometer (Thermo Fisher Scientific, Waltham, USA).

**CRATS procedure**

The one-pot CRATS reaction mixture was prepared by mixing two premixes, the RPA premix and the Cas12b premix. The RPA premix was prepared by adding 25 μL of A buffer and 4 μL each of the forward and reverse primers (10 μM) to the lyophilized enzyme pellet. The Cas12b premix contained 260 nM purified Cas12b, 2 μM molecular beacon, and 200 nM sgRNA in 1× NEB buffer 2.1. The 25-μL one-pot CRATS reaction mixture was made by mixing 14.5 μL of RPA premix, 6.25 μL of Cas12b premix, 1.25 μL of B buffer, and 3 μL of the sample. The reaction was carried out at 37°C for 20 min, followed by incubation at 60°C for 20 min in a mini metal bath. The FAM fluorescence signal was monitored with a Roche LightCycler 480 II qPCR machine. The endpoint fluorescence signal was observed by the naked eye under blue light.

**Samples**

The standard plasmid containing a monkeypox virus *F3L* gene fragment (GenBank No. ON568298/ON563414, nt 46168--46630) was purchased from Sangon Biotech Co., Ltd. (Shanghai, China). The pseudovirus samples representing monkeypox virus and other human-related orthopox viruses, including vaccinia virus, cowpox virus, variola virus, and buffalopox virus, and the DNA standards representing the SARS-CoV-2 N and ORF genes were purchased from Fubio Biological Technology Co., Ltd. (Shanghai, China). The genomic DNA of *Pseudomonas aeruginosa* (ATCC 9027) was obtained from ATCC. The skin swab samples were collected from consent-informed healthy individuals, immersed in 2 mL of virus storage solution (Beyotime Biotechnology, Shanghai, China), and separated into 200-µL aliquots. The simulated samples were prepared by mixing aliquots with the desired amounts of monkeypox pseudovirus. The copy number of the monkeypox pseudovirus in each simulated sample was quantified via real-time PCR. Briefly, the purchased monkeypox pseudovirus (10^8^ particles per mL) was tested with a Monkeypox Virus Nucleic Acid Detection Kit (qPCR method, BeNa Culture Collection Technology Research Center, Zhengzhou, China) to determine the correlation between the copy number and the *Ct* value. The simulated samples were subsequently tested with the same qPCR kit to confirm the copy number of each sample. DNA was extracted from the samples with a DNA Kit (Tiangen Biotech Co., Ltd., Beijing, China). The real-time PCR experiments were performed on a Roche LightCycler 480 II qPCR machine.

**Supplementary Table S1. Oligonucleotide sequences**

| Type | Description | Sequence (5′→3′) | Length (nt) |
| --- | --- | --- | --- |
| RPA primer | F3L-Forward | CGAGAAGTTAATAAAGCTCTGTATGATCTTCAACG | 35 |
|  | F3L-Reverse | CCTTATCGAATACTCTTCCGTCAATGTC | 28 |
| sgRNA | sgRNA-F3L | GUCUAGAGGACAGAAUUUUUCAACGGGUGUGCCAAUGGCCACUUUCCAGGUGGCAAAGCCCGUUGAGCUUCUCAAAUCUGAGAAGUGGCACCAGCUCCAACGAUACUCCUC | 111 |
| Molecular beacon | MB-FQ | FAM-TTATT-BHQ1 | 5 |
